# Lipid biophysics and/or soft matter-inspired approach for controlling enveloped virus infectivity

**DOI:** 10.1098/rsif.2021.0943

**Published:** 2022-04-13

**Authors:** Lamyaa Al-dalawi, Stephen P. Dunham, Cyril Rauch

**Affiliations:** School of Veterinary Medicine and Science, University of Nottingham, Sutton Bonington Campus, College Road, Sutton Bonington LE12 5RD, UK

**Keywords:** influenza viruses, soft matter physics, pulmonary surfactant, membrane biophysics, phospholipids

## Abstract

Proven as a natural barrier against viral infection, pulmonary surfactant phospholipids have a biophysical and immunological role within the respiratory system, acting against microorganisms including viruses. Enveloped viruses have, in common, an outer bilayer membrane that forms the underlying structure for viral membrane proteins to function in an optimal way to ensure infectivity. Perturbating the membrane of viruses using exogenous lipids can be envisioned as a generic way to reduce their infectivity. In this context, the potential of exogenous lipids to be used against enveloped virus infectivity would be indicated by the resulting physical stress imposed to the viral membrane, and conical lipids, i.e. lyso-lipids, would be expected to generate stronger biophysical disturbances. We confirm that when treated with lyso-lipids the infectivity three strains of influenza virus (avian H2N3, equine H3N8 or pandemic human influenza H1N1) is reduced by up to 99% in a cell-based model. By contrast, lipids with a similar head group but two aliphatic chains were less effective (reducing infection by only 40–50%). This work opens a new path to merge concepts from different research fields, i.e. ‘soft matter physics' and virology.

## 1. Background

Respiratory virus infections pose a significant threat to human health. Influenza viruses continue to cause recurrent epidemic and pandemic infections and the more recently emerged SARS-CoV 2 has threatened to overwhelm healthcare systems globally causing widespread mortality and disease. The human body has many natural or innate defences against viruses including pulmonary surfactant. Surfactant is composed of lipids (approx. 85% of phospholipids, approx. 10% neutral lipids) and proteins (approx. 5%) [1]. The initial physiological role and clinical importance discovered for pulmonary surfactant was its involvement in avoiding the collapse of pulmonary alveolus during end-exhalation [2]. More recently, pulmonary surfactant has been shown to participate in the innate host defence against respiratory pathogens [3–5]. Within the last decade, single lung surfactant phospholipids have been shown to protect, to some extent, cells from virus infectivity, including respiratory syncytial virus and influenza A [6–9]. Specific lipids found in the lung surfactant such as DPPG (di-palmitoyl-phosphatidylglycerol), POPG (palmitoyl-oleoyl-phosphatidylglycerol) or DPPC (di-palmitoyl-phosphatidylcholine) have been shown to antagonize virus infectivity [5,7]. However, a full understanding of their mechanism of action remains elusive.

The initial stage of replication requires the influenza virus to enter cells based on the interaction between viral surface hemagglutinin protein (HA) and cellular sialic acids. The stereochemistry and collective structure of viral HA, neuraminidase (NA) and matrix protein 2 (M2) are central for sucessful infectivity [10,11]. These proteins are embedded within a bilayer lipid envelope derived from cell membrane on budding.

Although it is often routine to think pharmacologically in terms of drug–target or surfactant component–virus chemical interaction, in the context of lipid surfactant the amphipathic nature of lipids, namely their property of being structurally composed of hydrophobic and hydrophilic parts, needs to be considered carefully.

Indeed, lipids have the unique property to self-assemble physically and it is perhaps not surprising then that in the presence of surfactant lipids, membrane blebs appear on the viral envelope [5]. Following Laplace's Law, without any variation in the osmotic pressure difference between the inside and outside of the virus, the formation of nanometric membrane blebs suggests a direct change in the surface tension of the viral envelope linked to the inclusion of surfactant lipids. As a result, it is possible that lung surfactant lipids interact directly with the bilayer membrane of the viral envelope in turn destabilizing the collective structure of key viral envelope lipoproteins, i.e. HA, NA and M2, with a resulting impact on virus infectivity. Such an effect would not be unexpected, as in mammalian cells the mechanical perturbation of the bilayer membrane linked to the exogenous inclusion of amphipatic materials, e.g. phospholipids, has been shown to affect the function of ion channels [12], drug transporters [13], endocytosis [14] and exocytosis [15].

Therefore, surfactant lipids may provide a biophysics-based generic immunity against a plethora of inhaled pathogens provided they have an outer bilayer membrane. If the physical properties of surfactant lipids are essential to set up this generic immunity, the next question to ask is: can we optimize them, i.e. what lipid surfactant would be the most efficient?

The basic geometry or topology of lipids is essential. Lyso-lipids, i.e. single aliphatic chain lipids, have a tendency to form small unilayer structures due to their conical shape whereas lipids with two aliphatic chains form larger bilayer liposomes due to their cylindrical shape [16]. The changes in the surface pressure generated upon inclusion into a bilayer membrane are expected to be different as lyso-lipids tend to promote sharper curvature.

Given the set of observations [5,17,18], the aim of this work was therefore to connect a physical intuition regarding the physical properties of lipids to virology. The current study is focused on biology/virology rather physics/modelling as its central purpose was to demonstrate, as a proof of concept, whether specific exogenous lipids reduce the infectivity of the influenza A viruses including H2N3, H1N1 and H3N8. Herein is shown that specific lipids are altering the virus morphology and inhibiting the binding of the virus to the host cell membrane with an impact on the expression of cellular cytokines (IL-8, TNF-*α*) and the expression of viral M-gene.

## 2. Results

### 2.1. Impact of exogenous lipids on H2N3 topology and structure

**Figure 1.**
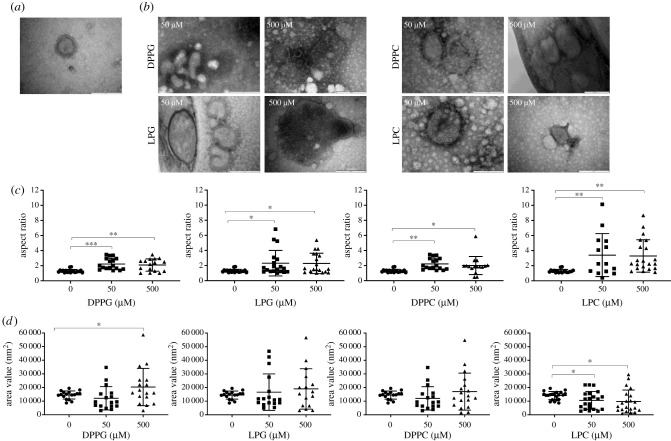
TEM images of H2N3 virus treated with phospholipids for 30 min at 37°C. (*a*) Control demonstrating spherical particles with clearly visible surface glycoprotein spikes. (*b*) H2N3 pre-treated with DPPG, LPG, DPPC or LPC at concentrations of 50 or 500 µM. Lipid vesicles are visible as white foci around the virus. Virions show altered morphology and rupture of the viral envelope is visible (scale bar: 200 nm). (*c*) The mean aspect ratio measurements changed significantly upon lipid pre-treatments indicating that virions showed a less spherical (aspect ratio of 1) and more elongated shape (aspect ratio greater than 1). (*d*) The mean surface areas of H2N3 pre-treated with phospholipids indicate that both larger and smaller virions are found after lipid treatment. These results confirm that phospholipids have an impact on virion morphology and that the impact generated is a function of the phospholipid used. (*p < 0.05; **p < 0.01; ***p < 0.001; one-way ANOVA.)

Hypothesizing that lipids have a physical impact on viruses, this should be visible using transmission electron microscopy (EM). Figure 1*a* shows a typical H2N3 virus where the surface glycoproteins and spherical shape of the virus are clearly visible. However, upon incubation with different classes of lipids including LPG, DPPG, LPC and DPPC for 30 min at 37°C, the virus morphology changed drastically as a function of concentrations used. EM pictures show that virions are surrounded by lipid structures visible as white foci next to the virus. Incubation of H2N3 with DPPC showed the least change in virion appearance, but at the other extreme, LPG treatment demonstrated rupture of the viral membrane suggesting an irreversible process (figure 1*b*). In order to quantify the impact of phospholipids on H2N3, the virus aspect ratio and surface area were measured (figure 1*c*,*d*, respectively) to document the observed change from a spherical shape toward a more elongated morphology. Uninfected H3N3 viruses showed a value for the aspect ratio of approximately 1 confirming that H2N3 are natively round (spherical). However, following treatment with amphipathic compounds, the mean aspect ratio increased demonstrating that viruses became ellipsoidal in shape. The change in aspect ratio was more pronounced following treatment with lysolipids (LPG or LPC). Regarding H2N3 surface area, results demonstrated a larger spread of numerical values for all lipids used (figure 1*d*). A larger surface area would be expected by the incorporation of exogenous lipids into to the virus membrane. However, virions with a reduced surface area, compared to untreated virus, were also observed, suggesting that upon incorporation of lipids, a virus may become unstable, undergoing fission to form smaller ‘daughter’ virions.

### 2.2. Minimal impact of exogenous lipids on mammalian cells

**Figure 2.**
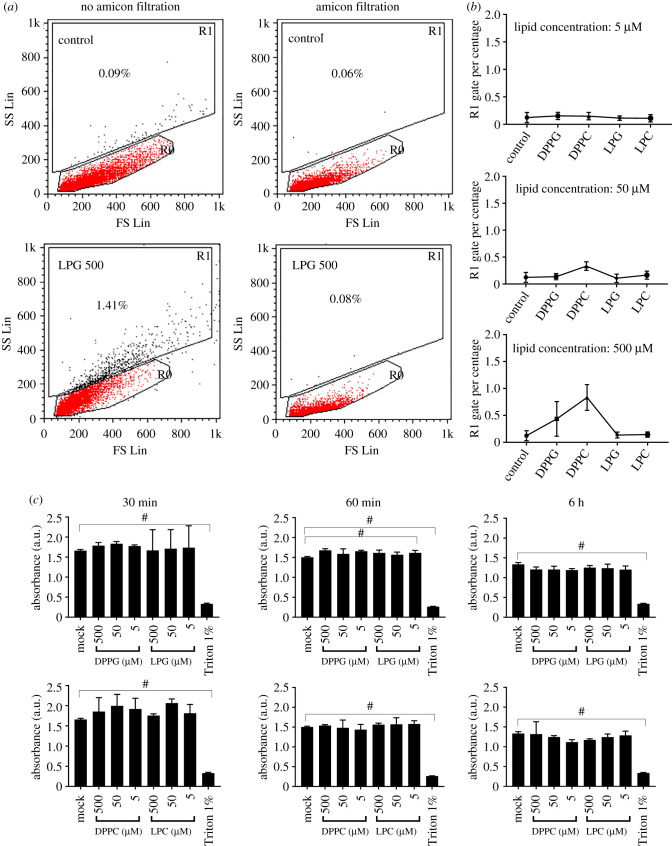
Impact of phospholipid on MDCK cells. (*a*) Incubation of LPG at a concentration of 500 µM without ultrafiltration (left panel) indicates an increase in the proportion of cells with increased side scatter (gate R1). This effect is minimized using ultrafiltration to remove excess lipid after virus treatment (right panel). (*b*) Percentage of cells with increased side scatter (R1 gated cells) is minimized after incubation with phospholipids at concentrations of 5, 50 and 500 µM followed by ultrafiltration. (*c*) Metabolic activity of MDCK cells treated with phospholipid solution which has been subjected to ultrafiltration; no significant reduction in cell metabolic activity was observed. Triton, used as a positive control, showed a significant reduction in cell metabolic activity. Means and standard errors of at least three experiments are represented. These results confirm that the phospholipids used have a minimal impact on cells. (*p < 0.05; **p < 0.01; ***p < 0.001; ^#^*p* < 0.0001; one-way ANOVA.)

Given the observation that viruses pre-treated with lipids demonstrated an irreversible impact on their morphologies, the direct impact of lipids on cells needed to be assessed as free lipids could also interact directly with cells used in virus infection experiments. Lipid solutions were prepared and incubated directly with Madin Darby canine kidney (MDCK) cells. Direct observation by light microscopy identified minimal differences in cellular morphology (data not shown). Flow cytometry was, therefore, used to assess the impact on cell morphology. The addition of 500 µM LPG to cells for 10 min resulted in a small proportion of cells (approx. 1.4%) showing an increase in side-scatter, suggesting increased cell granularity suggesting a change in the membrane intracellular architecture (figure 2*a*).

As a result, a method was devised to remove free lipids following the treatment of virus to minimize any direct impact on cells. After several optimizations using flow cytometry and measuring cell viability using an MTT essay, the best method found was to centrifuge the solution containing lipids using ultrafiltration. Indeed, the flow cytometry data demonstrated that once the retentate was resuspended with the infection medium only a small fraction (less than 0.1%) of MDCK cells were affected (figure 2*b*) and that their viability remained unchanged for a period of up to 6 h (figure 2*c*).

### 2.3. Impact of lipid pre-treatment on the attachment, internalization and infectivity of H2N3 in MDCK cells

**Figure 3.**
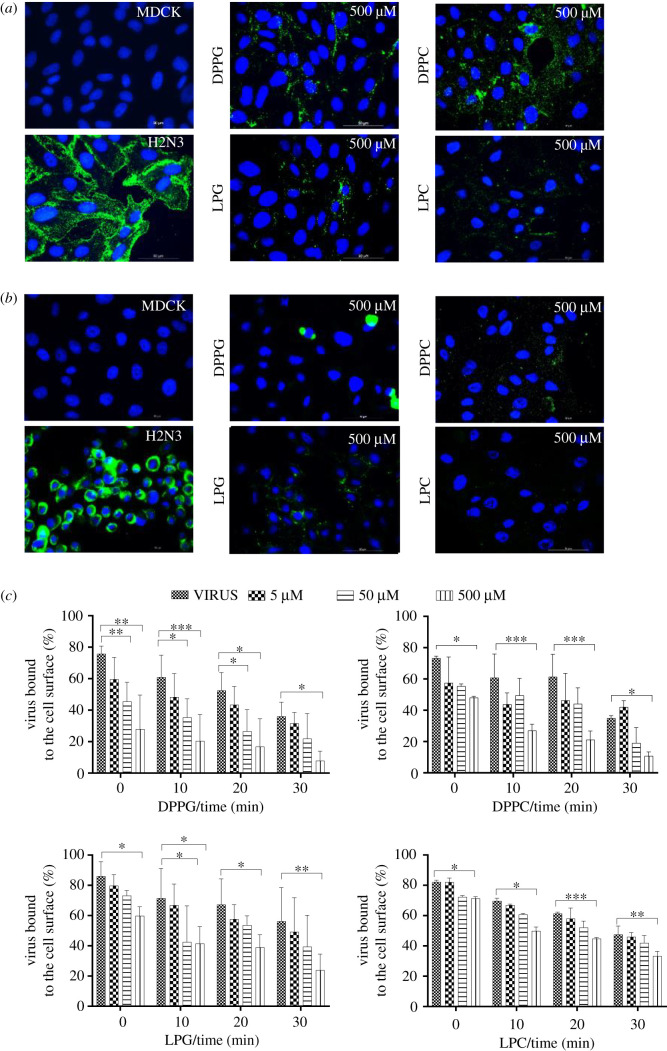
Influence of phospholipids on H2N3 (MOI 5) virus binding to MDCK cells. (*a*) Detection of H2N3 via FITC-labelled anti-HA antibody after binding at 4°C for 10 min and (*b*) after a further 30 min incubation at 37°C. (*c*) Flow cytometry analysis showing the proportion of cells with H2N3 bound to the surface over time. Means and standard errors of at least three experiments are represented. These results demonstrate that pre-treating H2N3 with phospholipids impacts on its ability to bind to cells. (*p < 0.05; **p < 0.01; ***p < 0.001; one-way ANOVA.)

As the lipids used had a strong impact on virus morphology it was hypothesized that such an impact could impair their ability to infect cells. To determine whether H2N3 virus attachment to cells was affected, virus (at a concentration to allow infection at a multiplicity of infection (MOI) of 5) was treated with lipids prior to ultrafiltration and the retentate was resuspended in cold infection medium (4°C). The cold infection solution was then incubated with cells for 10 min on ice. Cells were then washed with cold phosphate-buffered saline (PBS) and were either fixed with ice cold 1% PFA to determine the amount of viruses initially bound to cells, or reincubated with the infection medium at 37°C for different time points prior to fixation with PFA. HA was labelled using immunofluorescence without additional permeabilizing of MDCK cells to allow determination of the amount of viruses remaining on the MDCK surface over time. Figure 3*a* illustrates that the initial attachment of viruses to cells is impaired upon virus pre-treatment with lipids in comparison with untreated virus. After 30 min, only MDCK cells infected with untreated virus showed a noticeable diffuse fluorescent signal for HA suggesting that virus had entered cells and was detected by immunohistochemistry despite no additional permeabilization having been performed (figure 3*b*). However when the viruses were pre-treated with lipids a reduction in the fluorescent intensity within cells was clearly visible. In order to assess quantitatively the amount of viruses bound to the cell membrane over time flow cytometry was used. Data in figure 3*c* derived from flow cytometry analysis of MDCK infected cells demonstrate that the initial attachment of viruses to the MDCK cell membrane is impaired and is a function of the concentration of lipids used to pre-treat the virus.

**Figure 4.**
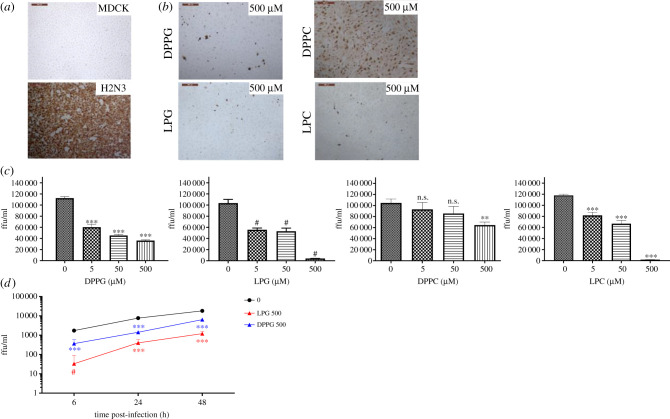
MDCK cells infected with H2N3 (MOI 1) with or without pre-treatment with phospholipids. (*a*) Immunocytochemical detection of influenza nucleoprotein in uninfected (MDCK) and infected (H2N3) cells 6 h post infection. (*b*) Immunocytochemical detection of influenza nucleoprotein in cells infected with H2N3 following pre-treatment with the indicated phospholipids and (*c*) with each lipid at 5, 50 or 500 µM resulting in a decrease in virus focus forming units that is concentration dependent. (*d*) Kinetics of infection for H2N3 untreated or following LPG or DPPG treatment 6 h, 24 h and 48 h post infection. Means and standard errors of at least three experiments are represented. These results demonstrate that while pre-treating H2N3 viruses with phospholipids impedes their ability to bind cells (figure 3), such pre-treatment does not impact on the ability of viruses that have been able to bind and enter cells to replicate inside cells and continue the infection. Thus, the pre-treatment of viruses with phospholipids delays the onset of infection. (*p < 0.05; **p < 0.01; ***p < 0.001; ^#^*p* < 0.0001; one-way ANOVA.)

The impact of lipid treatment on virus infection of MDCK cells was also assessed by immunofluorescent staining of cells for viral nucleoprotein. MDCK cells were infected with H2N3 virus at an MOI of 1.0 for 2 h, with or without lipid pre-treatment of virus inoculum, prior to washing cells in PBS and incubation of cells for a further 4 h. Cells were then fixed and the viral nucleoprotein (NP) was labelled using immunohistochemistry to determine the proportion of infected cells (measured as focus forming units (ffu) per ml). Data presented in figure 4*a*–*c* demonstrate that H2N3 infectivity was significantly reduced when DPPG, LPG or LPC were used for the virus pre-treatment as opposed to DPPC which showed little impact. LPG used at 500 µM showed the greatest reduction in virus infectivity (to 3.5% compared with untreated virus; table 1).

**Table 1.** Reduction in the percentage of virus infectivity for H2N3 in MDCK and A549 cells following DPPG or LPG treatment.

lipid	concentration (µM)	number of infected cells per field of view (*n* = 9) ± s.d. (% of virus infectivity)	
		H2N3/MDCK	H2N3/A549
virus only	NA	563 ± 53 (100%)	699.4 ± 84.7 (100%)
DPPG	5	301.3 ± 64.3 (53.5%)	392.5 ± 71.3 (56.1%)
	50	226.7 ± 69.3 (40.2%)	257.1 ± 47.5 (36.7%)
	500	181 ± 86 (32.1%)	109.0 ± 19.0 (15.5%)
LPG	5	278.7 ± 76.9 (49.5%)	305.6 ± 76.0 (43.7%)
	50	264.5 ± 71.1 (46.9%)	38.9 ± 19.4 (5.5%)
	500	20 ± 7.4 (3.5%)	3.1 ± 1.7 (0.4%)

However, with these data it was not possible to differentiate whether the drop in infectivity was simply linked to a reduction in the number of virions attached to cells initially or resulted from the impairment of the virus to multiply inside cells. To resolve this point, we studied the two lipids having the most significant impact on attachment and infectivity, i.e. LPG and DPPG. The infectivity experiment was then repeated with using virus at an MOI of 0.1 allowing infection to proceed to 48 h. Figure 4*d* demonstrates that when the virus was pre-treated with either LPG or DPPG the logarithmic increases of infectivity over time were similar to the control, in turn suggesting that the ability of H2N3 to multiply inside cells was not affected.

Taken together, the results suggest that a small fraction of pre-treated viruses are still functional and that pre-treating H2N3 with lipids only reduces the initial level of virus infection.

### 2.4. Transcription of IL-8, TNF-α and M-gene viral protein upon infection of MDCK cells with H2N3 pre-treated with lipids

**Figure 5.**
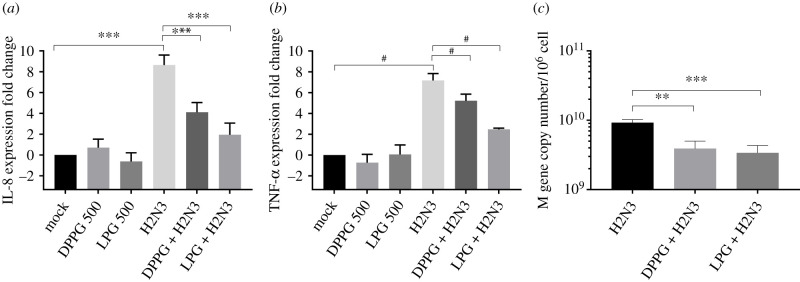
Cytokine transcription levels and M-gene copy number 24 h post infection of MDCK cells with H2N3 (MOI 5) pre-treated with 500 µM LPG or DPPG. (*a*) IL-8. (*b*) TNF-α. (*c*) M-gene copy number. Means and standard errors of at least three experiments are represented. Altogether those results confirm that the cells' response to infection is diminished when viruses are pre-treated with phospholipids. (*p < 0.05; **p < 0.01; ***p < 0.001; ^#^*p* < 0.0001; one-way ANOVA.)

In order to assess whether the cell responses were influenced by the reduced level of virus infection following lipid treatment, H2N3 was pre-treated with either LPG or DPPG and incubated with cells for 2 h prior to resuspending cells in a new infection medium (using untreated virus at an MOI of 5 as performed in figure 3*a*). After 22 h, cellular RNA was extracted and qRT-PCR used to determine the expression level of key host proinflammatory cytokines (IL-8 and TNF-α). Figure 5*a*,*b* demonstrates that the pre-treatment of H2N3 with either LPG or DPPG significantly reduced the expression of these cytokine mRNAs in MDCK cells. Cells treated with 500 µM of DPPG or LPG only, i.e. without viruses, showed no significant change in cytokine expression compared with untreated cells, suggesting that lipid treatment *per se* is not proinflammatory. Finally, the ability of H2N3 to multiply inside cells was also assessed by measuring the amount of mRNA for the viral M-gene by qRT-PCR. As expected the amount of M-gene mRNA expression was also downregulated when H2N3 viruses were pre-treated with lipids (figure 5*c*).

Altogether, these results confirm that pre-treating H2N3 with lipid reduces the level of virus infection, delays virus replication kinetics and consequently reduces the proinflammatory cell response.

### 2.5. Impact of lipid pre-treatment of H2N3 using A549 cells: attachment, infectivity and cell response

**Figure 6.**
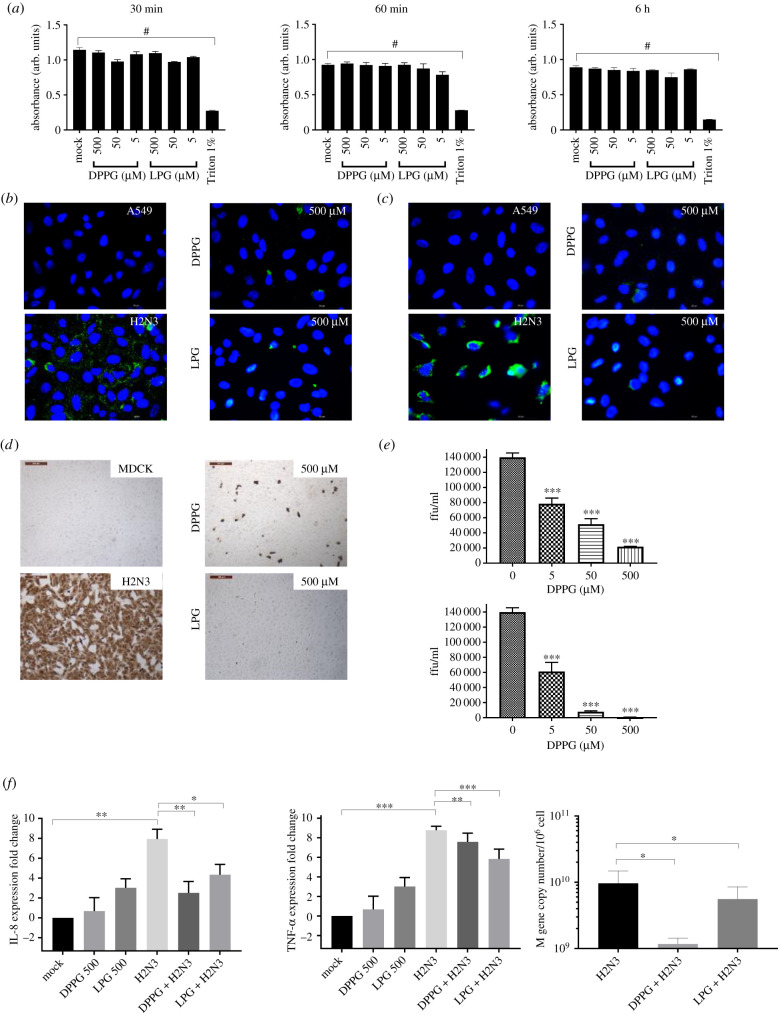
A549 cells infected with H2N3 upon following treatment with phospholipids. (*a*) Metabolic activity of A549 cells incubated with DPPG or LPG at different incubation time points following ultrafiltration showed no significant reduction in cell metabolic activity. Triton was used as a positive control. (*b*) Detection of H2N3 via FITC-labelled anti-HA antibody after binding at 4°C for 10 min and (*c*) after a further 30 min incubation at 37°C. (*d*) Immunocytochemical detection of influenza nucleoprotein in uninfected (MDCK) and infected (H2N3) cells 6 h post infection with or without lipid treatment and (*e*) with each lipid at 5, 50 or 500 µM resulting in a decrease in virus focus forming units that is concentration dependent. (*f*) Cytokine transcription levels of IL-8 and TNF-α from infected cells with lipid-treated H2N3 (MOI 1) with or without lipid treatment 24 h post infection. Means and standard errors of at least three experiments are represented. Those results demonstrate that the impact of phospholipid pre-treatment on H2N3 infectivity is not a function of the cell type used as similar trends are observed between cell types. (*p < 0.05; **p < 0.01; ***p < 0.001; ^#^*p* < 0.0001; one-way ANOVA.)

Our previous results demonstrated that H2N3 pre-treated with lipids impacts its ability to bind to and infect MDCK cells. However, such results could be specific to the cell line used. In order to assess this further, experiments were repeated using human adenocarcinomic alveolar basal epithelial (A549) lung cells. As previously determined with MDCK cells, the ultrafiltration method efficiently removed LPG and DPPG, with no impact on A549 cell viability noted using the MTT assay over a period of 6 h (figure 6*a*). Using the same method as for MDCK cells, H2N3 attachment to A549 cells was visibly impaired when H2N3 (sufficient to give an MOI of 5) was pre-treated with lipids (figure 6*b*,*c*). As expected, this resulted in a drop in virus infectivity, assessed as before, using immunohistochemistry for viral NP (at MOI of 1; figure 6*d*,*e*) that was more pronounced when LPG was used (infectivity reduced to 0.4% with 500 µM LPG or 5.5% with 50 µM LPG; table 1). IL-8 and TNF-α expression was similarly impacted by pre-treating H2N3 with either DPPG or LPG (figure 6*f*). Finally, it was found that DPPG and LPG significantly reduced H2N3 M-gene copy number, reflecting a reduction in cell infection (figure 6*f*). Those results confirmed that the virus ability to infect cells is altered upon treatment with lipids, irrespective of the cell line used.

### 2.6. Impact of lipid treatment on filamentous H3N8 virus

**Figure 7.**
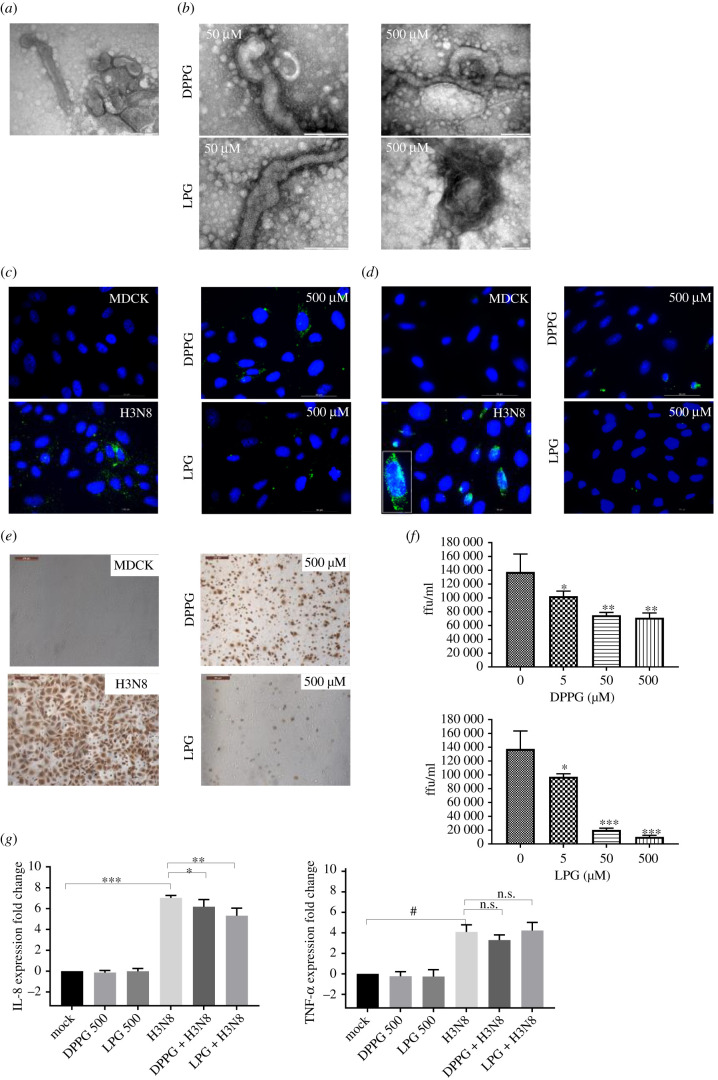
MDCK cells infected with equine influenza H3N8 following treatment with phospholipids. (*a*) TEM images of H3N8 virus without treatment show filamentous particles. (*b*) Phospholipid-treated H3N8 reveals observable changes in virus morphology including envelope rupture (scale bar, 200 nm). (*c*) Detection of H3N8 via FITC-labelled anti-HA antibody following virus binding (MOI 5) for 10 min at 4°C and (*d*) following further incubation for 30 min at 37°C. (*e*) Immunocytochemical staining of cells for virus NP 6 h post infection with H3N8 (MOI 1) or following treatment with LPG or DPPG and (*f*) with each lipid at 5, 50 or 500 µM resulting in a decrease in virus focus forming units that is concentration dependent (*p < 0.05; **p < 0.01; one-way ANOVA). (*g*) Cytokine transcription levels of IL-8 and TNF-*α* from H3N8 infected cells (MOI 1) with or without lipid treatment 24 h post infection. Means and standard errors of at least three experiments are represented. The results demonstrate that the infectivity of filamentous viruses is also affected by phospholipids. However, differences were observed and measured between spherical and filamentous viruses that may be related to the amount of membrane involved and the fact that the impact of lipids is diluted when the amount of membrane constituting the virus is larger. (*p < 0.05; **p < 0.01; ***p < 0.001; ^#^*p* < 0.0001; one-way ANOVA.)

Although H2N3 is natively spherical in shape, viruses can differ in their morphology and this is particularly the case for influenza viruses where filamentous viruses appear to be more frequently associated with clinical infections [19,20]. In order to assess the effect of lipid treatment on the infectivity of filamentous virus we used equine H3N8 which exhibits a largely filamentous morphology [21] to perform similar studies as for H2N3, using LPG or DPPG. Using EM, results demonstrated that treatment of H3N8 virus with either LPG or DPPG impacts its morphology (figure 7*a*,*b*), affecting the distribution of envelope glycoprotein. However, the reduction in infection elicited upon DPPG incubation was moderate when compared to greater reductions when H2N3 was used. Only when LPG was used were multilayered viral structures observed suggesting rupture of the viral membrane (compare figure 7*a*,*b* for LPG at 500 µM). Given the nature and three-dimensional shape of the filamentous virus it was not possible to quantify those changes through the measurements of H3N8 aspect ratio or its surface area using EM.

Regarding H3N8 attachment to cells, it was found that the interaction of the filamentous virus with MDCK cells was lower for untreated virus when compared to H2N3 in similar conditions (compare figure 7*c* with figure 3*a*), despite using virus at a similar MOI of 5. This may reflect a greater proportion of defective interfering particles in H2N3 virus preparation which are non-infectious but bind to MDCK cells. However, treatment with either LPG or DPPG still had a clear effect as the fluorescence intensity upon labelling H3N8 HA was much lower than the control (figure 7*d*). After a further 30 min incubation, infected cells were clearly visible for the control through the presence of clusters of fluorescence (figure 7*d*). Those clusters were not visible when H3N8 was pre-treated with either LPG or DPPG.

Measurement of infectious virus, by immunostaining for virus nucleoprotein, following incubation of virus for 30 min with LPG or DPPG (5, 50 or 500 µM) showed that virus pre-treatment with LPG had a more pronounced impact on H3N8 infectivity (MOI 1) when compared with DPPG (figure 7*e*,*f*). Treatment with 500 µM or 50 µM lipid reduced H3N8 infectivity to 7.4% or 14.7% for LPG and 52.0% or 54.4% for DPPG, respectively (table 2).

**Table 2.** Reduction in the percentage of virus infectivity for H2N3, H1N1 and H3N8 in MDCK cells following DPPG or LPG treatment.

lipid	concentration (µM)	number of infected cells per field of view (*n* = 9) ± s.d. (% of virus infectivity)		
		H2N3	H1N1	H3N8
virus only	n.a.	563.0 ± 53.0 (100%)	678.3 ± 35.4 (100%)	688.2 ± 165.0 (100%)
DPPG	5	301.3 ± 64.3 (53.5%)	485.7 ± 21.0 (71.6%)	517.1 ± 53.1 (75.1%)
	50	226.7 ± 69.3 (40.2%)	315.5 ± 41.5 (46.5%)	374.8 ± 36.1 (54.4%)
	500	181.0 ± 86.0 (32.1%)	31.3 ± 12.0 (4.6%)	356.4 ± 42.6 (52.0%)
LPG	5	278.7 ± 76.9 (49.5%)	521.0 ± 34.0 (76.8%)	486.4 ± 31.0 (70.7%)
	50	264.5 ± 71.1 (46.9%)	38.2 ± 10.6 (5.6%)	101.4 ± 15.5 (14.7%)
	500	20.0 ± 7.4 (3.5%)	9.2 ± 4.6 (1.3%)	51.2 ± 22.9 (7.4%)

Interestingly, it was found that only IL-8 transcription was significantly affected by pre-treatment of H3N8 (MOI 5) with either LPG or DPPG after 24 h infection, producing a modest reduction in the increased IL-8 expression observed following infection with untreated virus (figure 7*g*), whereas no significant changes were noted for TNF-*α*.

Those results suggest that the initial morphology of the virus, i.e. spherical or filamentous, has a significant impact on virus sensitivity to lipids. The impact of DPPG on virus infection was less marked when H3N8 was used when compared with H2N3. However, LPG had a greater impact on virus infection than DPPG for both viruses. Those results suggest that LPG is highly efficient whatever the initial morphology of the virus, but that the impact of DPPG is probably a function of the excess of viral membrane surrounding the virus, i.e. that the effect of such lipid is ‘diluted’ by the amount of lipid membrane forming the envelope of the filamentous virion. To confirm this, another spherical virus, H1N1, was used.

### 2.7. Impact of lipid pre-treatment on H1N1 infectivity and the transcription of IL-8 and TNF-α using MDCK cells

**Figure 8.**
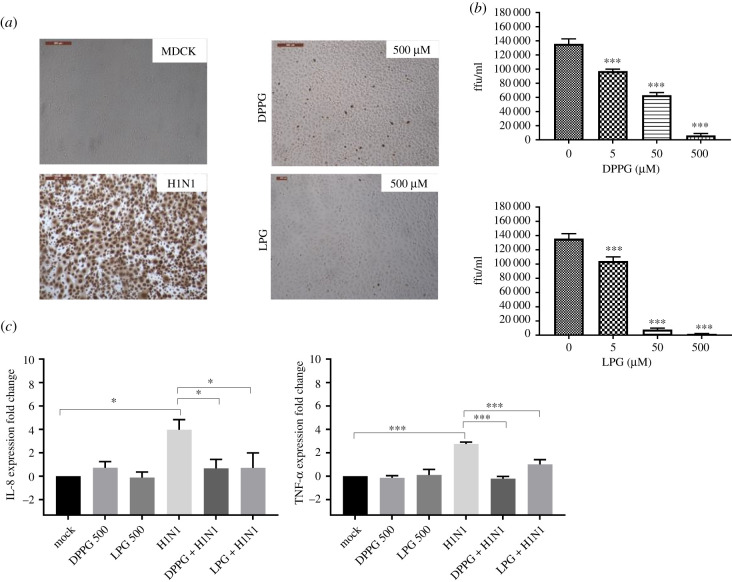
MDCK cells infected with influenza H1N1 following treatment with phospholipids. (*a*) Immunocytochemical staining of MDCK cells for virus NP, 6 h post infection with untreated H1N1 (MOI 1) or with H1N1 treated with phospholipids (500 µM DPPG or LPG) and (*b*) with each lipid at 5, 50 or 500 µM resulting in a decrease in virus focus forming units that is concentration dependent. (*c*) IL-8 and TNF-α mRNA expression in MDCK cells infected for 24 h with lipid-treated H1N1 (MOI 1) showed significant reduction in IL-8 and TNF-α transcription in comparison with untreated virus. Means and standard errors of at least three experiments are represented. Those results confirm that phospholipids have a generic impact on virus infectivity whatever the type of virus considered. (*p < 0.05; **p < 0.01; ***p < 0.001; ^#^*p* < 0.0001; one-way ANOVA.)

H1N1 (MOI 1) was pre-treated with DPPG and LPG using the same conditions as for H2N3 to measure its infectivity. Both LPG and DPPG altered H1N1 infectivity (figure 8*a*,*b*). The drop in infectivity for H1N1 treated with 500 µM DPPG to 4.6% was more pronounced than for H2N3 (32.1%; table 2). In line with the previous observations, LPG produced greater inhibition of virus infection compared with DPPG, resulting in a decrease to 1.3% or 5.6% infectivity when treated with 500 µM or 50 µM, respectively (table 2). Finally, the transcriptions of IL-8 and TNF-*α* were significantly and similarly reduced upon lipid pre-treatments of H1N1 (MOI 5) (figure 8*c*). This is consistent with the changes in IL-8 and TNF-*α* expression measured for H2N3 but differs from the results obtained using H3N8.

Tables 1 and 2 summarize the reduction in virus infectivity that was measured for each combination of lipid, virus and cell line.

## 3. Discussion

Influenza virus has a serious, worldwide, effect on human and animal health with a yearly seasonal death toll of many thousands, which increases during deadly pandemic episodes. Inactivated and attenuated vaccines against influenza A and B viruses (IAV and IBV) have been used to control influenza virus in addition to prophylactic and treatment attempts by using antiviral drugs but, still, significant research efforts are needed to control influenza viral infections. More recently the emergence of SARS-CoV 2 has resulted in an unprecedented virus pandemic that has had a devastating effect worldwide; mortality rates remain high and new treatments are urgently required.

The advances made in the field of soft matter physics encompassing lipid membranes and polymers have contributed to a better understanding of complex molecular structures and cell biology as a whole. The aim of this work was, therefore, to provide an interdisciplinary understanding of how lung surfactant phospholipids may function as an innate barrier against host pathogen infection, focusing on IAV. More precisely, based on the physical principle that single chain lipids are more prone to generate curvature upon their insertion into bilayer membrane, lipid surfactant analogues were tested against specific influenza viruses, i.e. H2N3, H3N8 and H1N1. The overarching conclusion is that lyso-lipids, in our case LPG and LPC, have indeed a more pronounced impact on virus infectivity independently of the cell type used (summarized in tables 1 and 2). It is remarkable that while concepts from soft-matter physics or biophysics have been successfully applied to a wide range of complex living systems allowing a better understanding of those systems, very little has been done in the field of virology.

In normal conditions H2N3 has a spherical shape showing outer glycoprotein spikes evenly distributed around the outer surface of the virus. In the presence of lipids, H2N3 is transformed into a giant/swollen structure and on some occasions, the viral envelope is ruptured. A less quantifiable but similar impact was observed on H3N8, a filamentous virus, suggesting that lipid interaction with the viral membrane is independent of the initial virus morphology. Previous studies have demonstrated that influenza virus binds efficiently with mixed liposomes composed of phospholipids within minutes [22] and that depleting the cholesterol from the viral envelope affects virus morphology, with virions showing nicks or holes in parts of the viral envelope [23]. Our study is therefore aligned with the latter conclusions that lipids interact with viruses and the envelope morphology is impacted upon changing its composition.

Those morphological alterations were associated with a decrease in the ability of viruses to interact with the cell membrane independently of the cell type. However, the ability of the virus to replicate inside cells was not affected, as the kinetics of replication were the same between the control and treated viruses. The influence of lipids on virus infectivity in the current study is in agreement with previous observations showing that the pulmonary surfactant phosphatidylglycerol (PG), which functions as the main innate immunity regulator in the lungs, blocks pulmonary viral infections [24] such as respiratory syncytial virus (RSV). This could provide an explanation for the higher concentration of phosphatidylglycerol in the lung compared to other body organs [7]. The airway epithelium is a primary barrier between the environment and the host, enhancing inflammatory and chemokine production targeting influenza viral infections and improving innate antiviral immunity [25]. Accordingly, and in agreement with a drop in infectivity, alterations in IL-8 and TNF-*α* cytokine transcription levels were measured together with a change in the number of mRNA copies for the viral M-gene 24 h post infection with influenza viruses.

Within the scope of this study our results demonstrate that pulmonary surfactant lyso-lipids can be used against H2N3, H3N8 and H1N1. The physical impact of lyso-lipids on viruses is expected to be the same across all enveloped viruses provided the presence of a bilayer membrane. While the use of lipids to treat acute respiratory distress has met with mixed results [26], the results presented here suggest that such treatments need to be rethought using optimized lyso-lipids with soft-matter/biophysical ‘functionalities’ such as to be properly effective against respiratory virus infections. Future treatments with lyso-lipids could therefore be used to control the ‘peak’ of infectivity for enveloped virus infections of the respiratory tract, including SARS-CoV 2. This could delay disease progression, and, in combination with other therapeutics, reduce disease morbidity and mortality.

In short, this paper is a call, or plea, to urge physicists to consider viruses as an important and central physical system to study.

## 4. Methods

### 4.1. Viruses

AI H2N3 (A/mallard duck/England/7277/06) and pandemic influenza A (H1N1) virus (A/California/7/2009) were kindly provided by Dr Ian Brown (Animal and Plant Health Agency (APHA), UK); a filamentous influenza, equine influenza H3N8 (A/equine/Newmarket/5/03), was kindly provided by Dr Debra Elton (Animal Health Trust, UK). Viruses were propagated in the allantoic fluid of embryonated hens' eggs. Virus titre was determined by immunocytochemistry as previously described [27] with aliquots stored at −80°C before use.

### 4.2. Cells

MDCK and A549 cells were grown and maintained in Dulbecco's modified Eagle's medium (DMEM, Glutamax; Invitrogen) containing 10% fetal calf serum (FCS; Invitrogen) and 1% penicillin/streptomycin (DMEM-10).

### 4.3. Lipids

Lipids 1,2-dipalmitoyl-*sn*-glycero-3-phospho-(1'-rac-glycerol) (DPPG); 1,2-dipalmitoyl-*sn*-glycero-3-phosphocholine (DPPC); 1-palmitoyl-2-hydroxy-*sn*-glycero-3-phospho-(1'-rac-glycerol) (LPG); 1-palmitoyl-2-hydroxy-*sn*-glycero-3-phosphocholine (LPC) (Avanti Polar Lipids, Inc.) were dried under nitrogen flow (Peak Scientific Instruments Ltd, UK) and resuspended in infection medium (IM) comprising serum free Ultraculture medium (Lonza, UK) supplemented with 1% penicillin/streptomycin (Invitrogen), 1% L-glutamine and 2 µg ml^−1^ tosyl phenylalanyl chloromethyl ketone trypsin (Sigma) prior to sonication at 60 kHz for 10 min to create a lipid emulsion.

### 4.4. Transmission electron microscopy

MDCK cells were infected with H2N3 or H3N8 at an MOI of 1.0 with incubation at 37°C with 5% CO_2_ for 72 h. The virus-containing supernatant was then harvested, clarified by centrifugation at 1200*g* for 2 min, and mixed with phospholipid solutions for 30 min at 37°C with 5% CO_2_. The final solutions were then pipetted onto holey carbon support film with 3.05 mm copper grids including 300 hexagonal mesh (EM Resolution, UK) and stained with 2% phospho-tungstic acid (PTA) at pH 7.4 (Sigma, UK). A Tecnai G212 Bio Twin Digital TEM system was used to image the samples from different areas of the grids and Image J (Image J 1.47v, National Institute of Health, USA) was used to measure virus aspect ratio and surface area to quantify virus morphology.

### 4.5. Flow cytometry to determine potential impact of lipids on MDCK cells

MDCK cells were cultured in DMEM-10 until 90–95% confluent. The medium was discarded and cells washed twice with cold PBS at 4°C. Cells were then incubated in versene (ethylenediamine-tetraacetic acid, Gibco, UK) at 37°C for 10 min to detach them. DMEM-10 was used to neutralize versene and the cell suspension was centrifuged at 1200*g* for 6 min. The pellet was resuspended at a concentration of 1 × 10^6^ cells ml^−1^ in IM. Single cell suspensions were then incubated with phospholipids at the desired concentration for 10 min at 37°C. To minimize the potential toxicity of phospholipids on cells, lipids were then removed by ultrafiltration using an Amicon Ultra-15 filter (50 K MWCO, Fisher Scientific, UK). Cells were then fixed in 1% PFA (Fisher, UK) for 20 min at room temperature and washed three times with cold PBS. The samples were then examined with an FC500 flow cytometer (Beckman Coulter, UK), recording 10 000 events, and the data analysed using Weasel software program v. 3.0 (Walter and Eliza Hall Institute, Australia).

### 4.6. Measurement of cell metabolic activity

MDCK or A549 cells were seeded in 96 well plates (Nunc) at 5 × 10^3^ cells well^−1^ with DMEM-10 at 37°C and 5% CO_2_ to reach 95% confluence. The supernatant was discarded and the cells washed three times with PBS prior to being incubated with 100 µl of phospholipid-IM solution at 37°C for 30 min, 60 min or 6 h. Triton 1% was used as a positive control. The cell metabolic activity was determined by using MTT reagent (CellTiter 96 Aqueous one solution for cell proliferation assay; Promega) following the manufacturer's instructions. Absorbance was read at 492 nm using a plate reader (Labtech International Ltd).

### 4.7. Flow cytometry to measure H2N3 binding and uptake using MDCK cells

H2N3 virus (at a concentration to allow infection at MOI of 5.0) was mixed with phospholipid solution at the desired concentration for 30 min at 37°C (5% CO_2_) prior to ultrafiltration to remove free lipids. MDCK cells were prepared as previously described and resuspended at a concentration of 1 × 10^6^ cells ml^−1^ in IM. Prepared cells and lipid-treated virus were then mixed together and incubated at 10 min at 4°C to allow virus binding, followed by 10, 20 or 30 min incubation at 37°C before centrifugation at 1200*g* for 2 min to recover the cell pellet that was then fixed using 1% PFA for 20 min at 4°C. Fixed cells were then blocked in 1% BSA (Fisher Scientific, UK) for 1 h at room temperature and incubated with specific chicken H3 antiserum (kindly provided by Ian Brown, APHA, UK) for 1 h at room temperature. After three washes with PBS, cells were incubated for 1 h with FITC-conjugated goat anti-chicken IGY (H + L) polyclonal secondary antibody (Novex, UK). Cells were then examined using the flow cytometer as described above.

### 4.8. Fluorescence microscopy to visualize H2N3 binding and internalization in MDCK and A459 cells and H3N8 binding and internalization in MDCK cells

MDCK or A549 cells were grown to 90–95% confluence on 13 mm glass coverslips and incubated with H2N3 or H3N8 (MOI 5) for 10 min at 4°C to allow virus binding, followed by 10, 20 or 30 min incubation at 37°C. Virus was incubated with phospholipid or IM prior to cell infection. Cells were in 1% PFA for 20 min at 4°C. Fixed cells were then blocked in 1% BSA (Fisher Scientific, UK) for 1 h at room temperature and incubated with specific chicken H3 antiserum for 1 h at room temperature. Cells were then incubated with Alexa Fluor 488 (goat anti-chicken IgY (H + L), Invitrogen). Then coverslips were mounted with Prolong Gold Anti-Fade Reagent with 4′,6-diamidino-2-phenylindole (DAPI; Life Technologies) and imaged using a Leica DM 5000B epifluorescence microscope.

### 4.9. Infectivity of influenza A virus pre-treated with phospholipids in MDCK cells or A549 cells

Influenza A viruses H2N3 and H3N8 were pre-treated with phospholipids at a range of concentrations. After ultrafiltration, as described above, the viruses were resuspended in infection medium and incubated with confluent MDCK cells (H2N3 or H3N8) or A549 cells (H2N3) at MOI of 1 for 2 h at 37°C. Cells were then washed three times with PBS at room temperature and incubated for a further 4 h in IM at 37°C before being fixed at 4°C in 1 : 1 acetone–methanol for 10 min at room temperature. Immunocytochemical staining was performed using a primary mouse monoclonal antibody (AA5H, Abcam, UK) for 1 h to detect influenza viral nucleoprotein which was then visualized with an Envision+ horseradish peroxidase phosphate (HRP) kit (DAB; Dako, Ely, UK) following the manufacturer's instructions. Finally, the proportion of cells stained for NP was calculated using counts from five fields captured using light microscopy assisted by Image J software, allowing calculation of focus forming units of virus per microlitre.

### 4.10. Influenza viral and host gene expression by quantitative RT-PCR

Viral RNA was extracted from cells using a QIAamp viral RNA purification kit (Qiagen) according to the manufacturer's instructions. The reverse transcription reaction of the viral RNA to cDNA was performed using a SensiFAST cDNA Synthesis Kit (Bioline) following manufacturer's instructions. Amplification of mRNA for IL-8, TNF*α* and IFN-β using human (for RNA extracted from A549 cells) or canine (for RNA extracted from MDCK cells) gene specific primers was performed using a SensiFAST SYBR No-ROX Kit (Bioline). 18S rRNA was used as a reference gene, previously shown to be suitable for analysis of cells infected with influenza A [28]. All primers were synthesized by Sigma Aldrich (table 3). Influenza Matrix gene (M gene) was quantified using a SensiFAST probe No-ROX Kit (Bioline). A forward primer (5′–3′) AGA TGA GTC TTC TAA CCG AGT CG and reverse primer TGC AAA AAC ATC TTC AAG TCT, with a hydrolysis probe (5′–3′) [6FAM]TCAGGCCCCCTCAAAGCCGA[BHQ1] were used to amplify a 101 bp region of the M-gene as previously described [29,30].

**Table 3.** Primers used for RT-PCR amplification.

gene name	forward primer (5′-3′)	reverse primer (3′-5′)	species
18S	TGTGCCGCTAGAGGTGAAATT	TGGCAAATGCTTTCGCTTT	human
18S	AGAAACGGCTACCACATCC	CACCAGACTTGCCCTCCA	canine
IL-8	GTTTTTGAAGAGGGCTGAG	TTTGCTTGAAGTTTCACTGG	human
IL-8	CTAAAGAAGGCTGAGAAAC	TTTGAAGTCTCATTGGCATC	canine
TNF-*α*	AGGCAGTCAGATCATCTTC	TTATCTCTCAGCTCCACG	human
TNF-*α*	TAGCTCATGTTGTAGCAAAC	AGTAGATGAGGTACAACCC	canine
IFN-β	CCAGTTCCAGAAGGAGGACA	TGTCCCAGGTGAAGTTTTCC	canine

### 4.11. Statistical analysis

Statistical analyses were performed using GraphPad Prism Student (v. 6.07). A one-way ANOVA with Tukey's multiple comparisons test was used to determine significant differences between different treatments. Differences were considered significant for *p* < 0.05.

## Data Availability

Data are available upon request. Data are available from the electronic supplementary material [31].
